# Role of Non-Neuronal Cells in Body Weight and Appetite Control

**DOI:** 10.3389/fendo.2015.00042

**Published:** 2015-03-26

**Authors:** Pilar Argente-Arizón, Alejandra Freire-Regatillo, Jesús Argente, Julie A. Chowen

**Affiliations:** ^1^Department of Endocrinology, Hospital Infantil Universitario Niño Jesús, Instituto de Investigación La Princesa, Madrid, Spain; ^2^Department of Pediatrics, Universidad Autónoma de Madrid, Madrid, Spain; ^3^Fisiopatología de la Obesidad y Nutrición (CIBERobn), Centros de Investigación Biomédica en Red, Instituto de Salud Carlos III, Madrid, Spain

**Keywords:** glia, ependymal cells, high fat diet, leptin, ghrelin, metabolism, hypothalamus

## Abstract

The brain is composed of neurons and non-neuronal cells, with the latter encompassing glial, ependymal and endothelial cells, as well as pericytes and progenitor cells. Studies aimed at understanding how the brain operates have traditionally focused on neurons, but the importance of non-neuronal cells has become increasingly evident. Once relegated to supporting roles, it is now indubitable that these diverse cell types are fundamental for brain development and function, including that of metabolic circuits, and they may play a significant role in obesity onset and complications. They participate in processes of neurogenesis, synaptogenesis, and synaptic plasticity of metabolic circuits both during development and in adulthood. Some glial cells, such as tanycytes and astrocytes, transport circulating nutrients and metabolic factors that are fundamental for neuronal viability and activity into and within the hypothalamus. All of these cell types express receptors for a variety of metabolic factors and hormones, suggesting that they participate in metabolic function. They are the first line of defense against any assault to neurons. Indeed, microglia and astrocytes participate in the hypothalamic inflammatory response to high fat diet (HFD)-induced obesity, with this process contributing to inflammatory-related insulin and leptin resistance. Moreover, HFD-induced obesity and hyperleptinemia modify hypothalamic astroglial morphology, which is associated with changes in the synaptic inputs to neuronal metabolic circuits. Astrocytic contact with the microvasculature is increased by HFD intake and this could modify nutrient/hormonal uptake into the brain. In addition, progenitor cells in the hypothalamus are now known to have the capacity to renew metabolic circuits, and this can be affected by HFD intake and obesity. Here, we discuss our current understanding of how non-neuronal cells participate in physiological and physiopathological metabolic control.

## Introduction

Non-neuronal cells, which include glia, ependymal and epithelial cells, and pericytes, outnumber neurons in the central nervous system (CNS); however, their functions have been less well studied. It has become increasingly clear during the past two decades that these diverse cells are not only vital for neuronal support and survival, but that they are also active participants in brain development and function (1–4). Increasing our knowledge of how non-neuronal cells function will not only lead to a better understanding of normal brain physiology, but it could also shed light on specific physiopathological processes and result in the identification of new therapeutic targets.

During the past decade, the interest in how non-neuronal cells participate in the neuroendocrine control of metabolism has escalated. This increased attention is due, at least in part, to studies demonstrating that glial cells are intimately involved in producing the hypothalamic inflammation that results from high fat diet (HFD)-induced obesity, and that is linked to increased insulin resistance (5–8). Indeed, glial cells are now known to participate in diverse pathological processes associated to excess weight gain (6, 9–12). In addition to being involved in the physiopathological responses to poor dietary habits and obesity, non-neuronal cells also play a comprehensive role in the physiological neuroendocrine control of metabolism.

Glial cells are generally classified into microglia and macroglia, with the latter including astrocytes, tanycytes, oligodendrocytes, and ependymal cells. In order to understand how these cells can affect metabolic circuits, it is important to have a basic understanding of their known functions in the CNS. In addition, new functions of epithelial cells and progenitor cells in the renovation of metabolic circuits have been uncovered in recent years. Here, we briefly review the general functions attributed to these cells and then what is currently known regarding their interaction with central metabolic circuits. Special emphasis is placed on glial interactions with hypothalamic neuropeptide Y (NPY) and proopiomelanocortin (POMC) neurons, as these neuronal populations are fundamental for the control of appetite and satiety.

## Microglia

Microglia are the primary immunological cells of the CNS. Unlike other glial cells, which are derived from the neuroectoderm, microglia are of mesodermal origin, and unlike other immunological cells, microglial populations are normally maintained by self-renewal and do not depend on myeloid progenitors (13). Although this self-renewal hypothesis is generally accepted, in some cases monocytes are reported to contribute to modifications in microglial populations (14).

Three subtypes of microglia can be distinguished according to their morphology: (1) Amoeboid microglia, which are a transitory form linked to development that disappears in the early postnatal period; (2) Ramified or resting microglia; and (3) Reactive microglia (15, 16). Although each microglial subset is thought to perform specific functions, these morphological/functional relationships have not been thoroughly characterized (17). Moreover, the concept of resting versus reactive microglia is now being challenged, as microglial activation appears to be a continuous spectrum of phenotypes and functional states that range from protective to harmful.

## Microglia in Health and Disease

**Figure 1:**
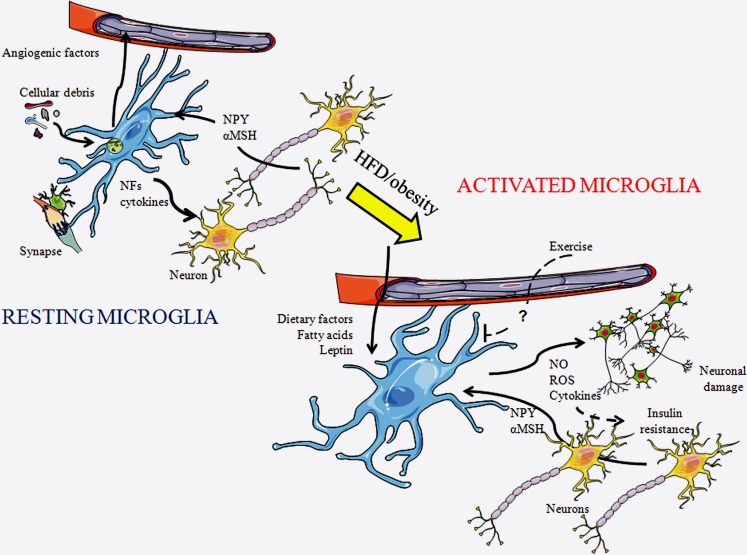
**Schematic representation of microglial cells in a resting state and in an activated state**. In a resting state, microglia are constantly patrolling the local environment to detect abnormalities or invading substances. They clean-up cellular debris, modulate synapses, and produce diverse substances including cytokines. As microglia become activated their morphology changes, with their projections becoming shorter and thicker. In response to a HFD or obesity, diverse factors reach the central nervous system through the circulation and can activate microglia. These cells produce diverse factors, including inflammatory cytokines, reactive oxygen species (ROS), and nitric oxide (NO), which further activates local microglia and can damage neurons. This process can lead to insulin resistance. Exercise is known to inhibit this process, although the factors mediating this remain unknown. NFs, neurotrophic factors; αMSH, α-melanocyte stimulating hormone; and NPY, neuropeptide Y.

Microglial cells participate in a number of fundamental processes both during development and in adulthood (Figure 1). Microglia are continuously changing their shape and sending-out processes. This allows them to move about and examine their surroundings in order to spot irregularities and deviations from homeostasis, to which they can then respond accordingly. One important function is the phagocytosis of cellular debris, damaged cells, plaques, and foreign matter (18). Some microglial subtypes are able to release nerve growth factors, neurotrophins, and other neurotrophic factors to provide support to neurons (19). Microglia colocalize with dying neurons during development (20–24), and this is suggested to indicate that they are involved in the regulation of neuronal number and development. These glial cells are also reported to promote angiogenesis during development and to be activated in response to damaged vessels (20, 25–27).

Microglia can modulate neuronal activity through the promotion of synaptic plasticity and the release of neurotrophic factors and anti-inflammatory cytokines (28–30). They also take part in “synaptic stripping” or the removal of branches from damaged neurons (31–33). A quad-partite synapse model, where microglia play a dynamic role in neural communication in association with astrocytes, has recently been proposed (34), but further study is necessary to consolidate this concept. When performing these physiological functions, microglia may exhibit a morphology that is classically characterized as activated; however, their functional aim cannot be classified as a pro-inflammatory response. Thus, microglial function is not always implied by their phenotype.

When microglia are activated in response to brain injury or foreign substances, they release cytotoxic factors in order to destroy or neutralize the invading pathogen or toxin. They can also participate in the presentation of antigens to T-cells during active infections (18). Moreover, it is now known that these glial cells can be activated in response to specific nutrients, such as saturated fatty acids (FAs) (35).

## Microglia in Metabolic Control

Reactive microgliosis is triggered by an initial assault or neuronal damage. This process is then propelled due to the release of substances, both by reactive microglia and damaged neurons, which activate more microglia, with this gliosis ultimately resulting in neurotoxicity (36). HFD-induced obesity induces chronic low-grade hypothalamic inflammation that involves activation of both microglia and astrocytes. This inflammatory process eventually leads to neuronal injury, with POMC neurons being especially vulnerable (8). As POMC neurons stimulate energy expenditure and reduce appetite, long-term microgliosis could possibly perpetuate weight gain through inflammation-induced damage to these anorexigenic neurons. Microgliosis can be found in appetite-regulating areas of the hypothalamus in response to HFD intake, even without significant weight gain (8), suggesting a direct effect of the diet itself. Indeed, as mentioned above, saturated FAs can trigger reactive microgliosis (35). Moreover, the inflammatory effect of FAs is specific as saturated FAs activate microglia, while monounsaturated FAs do not (35). The microglial response to saturated FAs is also anatomically specific, being found in the hypothalamus, but not in other brain areas (8, 35), which could be related to this region’s role in FA metabolism and nutrient sensing. However, this deserves further study, as the microglial response has not been analyzed in all areas involved in metabolic control. Some neuropeptides involved in metabolic control, such as NPY and α-melanocyte stimulating hormone (αMSH), which is released from POMC neurons, can directly modulate the production of cytokines and nitric oxide (NO) by microglia (37–39), indicating a direct link between these neurons and microglia that could also contribute to inflammatory processes.

Circulating metabolic hormones are also involved in obesity-induced microglial activation. Leptin increases in proportion to fat mass and can directly activate microglia (6, 40), suggesting that this hormone mediates some of the effects of increased adiposity on these glial cells. In support of this, Gao et al. (41) recently revealed that increased body weight, diet, and leptin levels interact to determine microglial activity. They reported that ob/ob mice, which are very obese due to the lack of leptin and its anorexigenic effects, have lower levels of hypothalamic microglial activation than control mice. Although HFD intake increases hypothalamic microglial activation in these mice, this activation does not reach control levels. Leptin treatment increases microglia number and ramification in ob/ob mice, even when associated with weight loss (41). This reinforces the concept that it is not body weight itself, but the interaction of hormones and dietary signals that control hypothalamic microglial activity during obesity.

Central inflammation is linked to the pathogenesis of insulin resistance and type 2 diabetes (42). Microglial activation results in the release of pro-inflammatory cytokines that can activate the intracellular signaling pathways that lead to insulin resistance and ultimately to type 2 diabetes (5). Central inhibition of the inflammatory pathways C-Jun N-terminal kinase and nuclear factor-kappaB improves insulin resistance in obese animals, with inhibition of inflammatory signaling specifically in AgRP neurons protecting against obesity and glucose intolerance (42, 43). Interestingly, exercise reduces microglial activation caused by HFD, and this also results in improved glucose tolerance (44). This suggests that exercise is beneficial not only because it provokes increased energy expenditure but also because it improves central inflammatory processes. Thus, microglial activation and hypothalamic inflammation appear to be of great importance in both the perpetuation of weight gain and the development of secondary pathologies associated with obesity.

## Microglia in the Long-Term Metabolic Effects of the Early Nutritional Environment

The nutritional environment during early life can modulate the propensity to gain weight and develop secondary pathologies in later life, and it also influences the maturation of immune cells. Indeed, the type of nutrition during early life is suggested to have lasting effects on the response to subsequent neuroimmune challenges (45, 46). Microglia present a prolonged “primed” or sensitized status when their normal pattern of maturation is disrupted, and this affects later central immune function (47, 48).

In a primate model of HFD intake during gestation, the release of cytokines by activated hypothalamic microglia was shown to influence the development of hypothalamic melanocortin circuits in the fetus (49). In the offspring of HFD-fed rodent mothers, an increase in hippocampal microglial activation at birth and in microglial density in adulthood has been reported (48). Rats with neonatal overnutrition due to being raised in small litters are overweight in adult life even when they receive a regular chow diet (11). These rats have increased microglial activation in specific hypothalamic nuclei (11) and other brain regions (11, 50) and increased expression of inflammatory genes in the adult hypothalamus (51). In addition, their microglial response to subsequent stresses, such as lipopolysaccharide endotoxins, is enhanced (50, 51). The activated microglial profile of neonatally overfed rats may also affect neurogenesis, cognitive function, and behavior (52). Although there are significant sex differences in microglial number and morphology in various brain regions (53), neonatal overnutrition is reported to induce similar changes in microglia in the hypothalamic paraventricular nucleus (PVN) of both males and females (51). Thus, poor dietary habits during early life can have long-term effects on both the central immune system and the neuroendocrine control of metabolism, with the interaction of these two processes most likely attributing to the increased sensitivity to obesity-associated pathologies.

## Astrocytes

Astrocytes are the most abundant and diverse non-neuronal cell type in the CNS. There are approximately five times more astroglia than neurons, although this proportion varies from one area of the brain to another (54). These macroglial cells originate from ectoderm (55) and are known as “the star-shaped cells” due to their appearance when visualized by conventional immunolabeling techniques. They have a privileged location throughout the brain as they form part of the blood–brain barrier (BBB) and are in intimate contact with both vascular and synaptic elements (56, 57), making them essential components in the communication between the CNS and periphery (58). One important function of these glial cells is to provide blood-borne nutrients and other essential substances to neurons, thus promoting neuronal survival and contributing to the maintenance of CNS homeostasis. The astrocytic endfeet that surround blood vessels express specific membrane proteins, such as glucose transporters (GLUTs), in order to transport energy substrates from the circulation into the brain (59), and they also secrete substances that can modulate local blood flow (60, 61). In conjunction with pericytes, these astrocytes contribute to the maintenance of BBB homeostasis (62–64).

One of the classical roles attributed to astrocytes is to provide anatomical support for neurons. They also regulate neuronal differentiation, proliferation, and synaptogenesis during development (2, 65). Astrocytes occupy specific non-overlapping domains, with each glial cell contacting various neurons and thousands of synapses (66–68). Moreover, they form a syncytium that allows them to regulate neuronal and synaptic function over long distances (69, 70) by communicating with each other via a network of gap junctions (71, 72).

Astrocytes modulate synaptic transmission in various ways, including the re-uptake of glutamate from the synaptic cleft, which terminates excitatory transmission and protects against excitotoxicity, and by participating in synaptic reorganization (1, 2, 73, 74). They also actively participate in tripartite synapses by secreting gliotransmitters, such as adenosine, ATP, D-serine, glutamate, and TNFα, to modulate synaptic efficacy (75–82).

Like microglia, astrocytes take part in central immune responses by becoming activated and producing cytokines in response to infections, foreign substances, and CNS injuries (83–86). This protective response can vary widely, spanning from acute activation that involves changes in astrocyte morphology and proliferation to scar tissue formation associated with secretion of inflammatory cytokines, which can then activate the NFκβ pathway and stimulate further inflammatory cytokine release (87). Depending on the type of stimulus, its intensity, and the time of exposure, the cytokines secreted by astrocytes can have either beneficial neuromodulatory effects or detrimental inflammatory effects.

Due to their wide array of functions, astrocytes are fundamental for neuronal survival throughout the entire brain; however, these glial cells are heterogeneous with their morphology, density, and activational responses differing from one anatomical area to another (88, 89). Moreover, astrocytes differ between males and females, with this sexual dimorphism being partially due to differences in neonatal levels of sex steroids, in addition to postpubertal hormones (86, 90–93).

Astrocyte morphology is generally classified as either protoplasmic or fibrous. Protoplasmic astrocytes have short divided projections and are predominately found in the gray matter, close to neuronal synapses and blood vessels. In contrast, fibrous astrocytes are located within the white matter, and their processes are long and relatively unbranched (94, 95). The diversity of astrocyte morphology was appreciated for the first time in the drawings by Cajal (96). In order to detect and identify astrocytes, the intermediate filament protein glial fibrillary acidic protein (GFAP) has been extensively used (54, 97, 98). However, many astrocytes are not GFAP positive, which is an important limitation for their study. The search for both global and functional markers of astrocytes is ongoing, and the future use of these new tools will most likely lead to the discovery of additional astrocytic functions.

**Figure 2:**
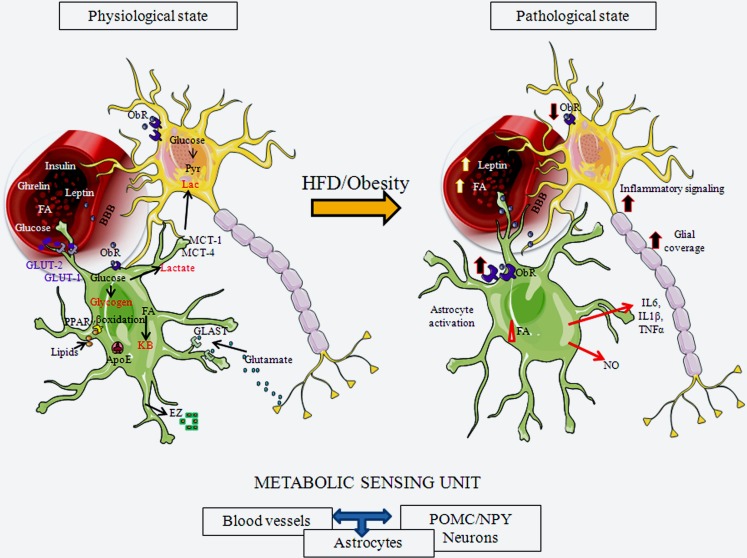
**Schematic representation of hypothalamic astrocytes in a normal physiological state and in a pathological state in response to exposure to a HFD or obesity**. Astrogial cells express receptors for important metabolic hormones, such as leptin and insulin, and transport diverse substances, including glucose, from the circulation into the brain. They can store glucose as glycogen or metabolize it to lactate, which can be secreted for uptake and usage by neurons. Astrocytes also metabolize lipids. They participate in normal glucose and lipid sensing to control energy homeostasis. Astrocytes participate in synaptic transmission through the uptake of glutamate from the synaptic cleft. Upon activation by a HFD or obesity, these physiological processes can be altered by both nutritional signals, such as FAs, and metabolic signals, such as leptin. Astroglial morphology changes, with these cells increasing their projection number and size and, increasing the glial coverage of both POMC and NPY neurons, as well as their contact with local blood vessels. This results in modifications in synaptic inputs to these neurons and possible changes in transport of substances from the circulation. These activated astrocytes also secrete cytokines and other factors, participating in activation of inflammatory signaling in neurons. ApoE, apolipoprotein E; BBB, blood-brain barrier; EZ, endozepines; FA, fatty acids; GLAST, glutamate aspartate transporter; GLUT-1, glucose transporter 1; GLUT-2, glucose transporter 2; KB, ketone bodies; IL6, interleukin 6; IL1β, interleukin 1 β; MCT-1, monocarboxylate transporter 1; MCT-4, monocarboxylate transporter 4; NO, nitric oxide; ObR, leptin receptor; PPARγ, peroxisome proliferator-activated receptor gamma; TNFα, tumor necrosis factor α.

It should be noted that even within the hypothalamus, astrocytes are regionally quite distinct, although there is still little information regarding the functional significance of these differences (99). Neuroendocrine functions of hypothalamic astrocytes were described more than three decades ago (100); however, analysis of their role in systemic metabolic control is more recent. The rapid rise in obesity and its comorbidities has contributed to the increased drive to understand the neuroendocrine mechanisms controlling metabolism. As a result, the contribution of astroctyes to the physiological and pathophysiological control of metabolism has come to the forefront. Their participation in diverse processes related to metabolic control is represented in Figure 2.

## Astrocytes in Glucose and Fatty Acid Sensing

Glucose is the major energy source for the mammalian brain, and astrocytes are not only involved in actively transporting glucose from the circulation into the brain, but they are also in charge of its storage. The astrocytic endfeet that enclose capillaries express GLUT-1 in order to transport glucose into the CNS (59, 101, 102). This glucose is then either stored inside astrocytes as glycogen or metabolized. Astrocytes can metabolize glucose to lactate, which is then shuttled to neurons to be used as an energy source, with studies showing that increased glucose transport/metabolism is coupled to increased neuronal activity (103).

Due to the privileged location of astroglial cells, they are among the first cells in the CNS to perceive peripheral glucose concentrations. Hypothalamic astrocytes and neurons act together to sense circulating levels of glucose in order to “inform” the brain about the systemic metabolic condition. This glucose sensing process is important in order to maintain both central and systemic glucose homeostasis and can thus generate modifications in feeding behavior (104).

In brain areas involved in appetite control, astrocytes express high levels of GLUT-2. This GLUT is essential for glucose sensing and for the homeostatic control of circulating glucose levels and food intake (105, 106). Indeed, food consumption is increased in transgenic mice expressing a form of GLUT-2 that is unable to detect glucose levels, but maintains its ability to transport glucose (106). Although expression of GLUT-2 in astrocytes is fundamental for normal glucose sensing, neuronal glucose sensing is suggested to be mediated through another mechanism (105). Moreover, astrocytes (and tanycytes) in the arcuate nucleus secrete endozepines, which not only have strong anorexigenic actions (107), but also participate in glucose sensing, possibly through the melanocortin pathway (108). In addition, it was recently shown that they also participate in the regulation of long-chain fatty acids (LCFA) metabolism in astrocytes (109).

Energy homeostasis is regulated by the ability of the hypothalamus to correctly sense FA levels and to metabolize this fuel source (110, 111). Neurons, astrocytes, and oligodendrocytes can use glucose and ketone bodies as energy substrates, but only astrocytes can β-oxidize FAs to produce ketone bodies (112), with astrocytes known to be the main source of FA oxidation in the brain (104, 113). Taïb et al. (114) demonstrated that FA oxidation rates are dependent on glucose concentrations, with glucose inhibiting this process in hypothalamic astrocytes via activation of AMPK. Astrocytes also express peroxisome proliferator-activated receptor gamma (PPARγ), an important sensor of lipids and regulator of metabolism (115, 116), and apolipoprotein E (ApoE), the major lipid transporter in the CNS (117, 118). ApoE can also act as a satiety factor, possibly by mediating some of the inhibitory effects of leptin (119), and may play a protective role against apoptosis of astrocytes (120).

The ketone bodies produced as a result of FA oxidation by astrocytes serve as an energy source for neurons, especially when neuronal activity is intense or in situations of hypoglycemia, such as in fasting (113, 121, 122). However, ketone bodies are also synthesized by astrocytes during chronic HFD, and this can disrupt FA signaling mechanisms involved in metabolic control (123). As astrocytes are fundamental for central FA metabolism, it follows that these glial cells participate in hypothalamic FA sensing and its subsequent effects on appetite.

## Astrocytes Express Receptors for Metabolic Hormones

Astrocytes express receptors for hormones involved in energy balance, such as leptin, insulin, glucocorticoids, and ghrelin (124–128), and may mediate some of the metabolic effects of these hormones at the level of the hypothalamus. How leptin modulates astrocytes has been the subject of diverse studies in recent years. Hypothalamic astrocytes express different isoforms of the leptin receptor (ObR), expressing mainly ObRa and ObRb, but also including ObRc and ObRe (127, 129–132). In the hypothalamus of obese rodents, expression of this receptor is increased in GFAP positive cells (127, 129–132), and its expression in astrocytes varies between brain areas, with the highest expression being found in the hypothalamus (10).

It is possible that leptin modulates its own transport into the CNS, as well as the nutrient supply to neurons, by acting on astrocytes (133, 134). Moreover, astrocyte morphology is changed by leptin (10, 132), and this can affect synaptic inputs to POMC and NPY neurons, and thus their function (132). Over a decade ago, leptin and ghrelin were shown to rapidly modulate the number of synaptic inputs onto hypothalamic POMC and NPY neurons (135–137), and changes in glial morphology are most likely involved in this process. Indeed, in obese and control animals, the number of synapses on the soma of hypothalamic POMC neurons is inversely related to their glial coverage, with increased glial ensheathment leaving less neuronal membrane available for synaptic inputs (9). The rapid modifications in synaptic inputs to metabolic neuronal circuits produced by leptin, as well as in response to HFD intake, most likely represent an adaptation of the system to the new metabolic condition (138) and an attempt to maintain metabolic homeostasis. However, if the exposure to increased levels of leptin or HFD is prolonged, these responses may become pathophysiological. Indeed, the glial response to leptin is time dependent, and prolonged exposure to this hormone stimulates astroglial production of different cytokines (10), as discussed below.

Astrocytes express high levels of the glutamate transporters GLT-1 and GLAST (139–141) through which they regulate glutamate levels in the synaptic cleft. This process is essential for maintenance of appropriate synaptic transmission and for the prevention of excitotoxicity and cell death. Once glutamate is taken-up by astrocytes it can be converted to glutamine by the enzyme glutamine synthetase, which is almost exclusively expressed in astroglia (142). Glutamine is then shuttled to neurons to produce more glutamate, or GABA, for synaptic release. Glutamate can also be metabolized in astrocytes and converted to α-ketoglutarate (143, 144).

There is a tight relationship between glutamate uptake, glycolysis, and lactate production such that when neuronal demand is higher, glucose transport and utilization by astrocytes increases, and in consequence, more lactate is produced to be used as an energy substrate by neurons (145, 146). Leptin modifies the ability of hypothalamic astrocytes to transport both glutamate and glucose, with the time of leptin exposure producing different responses (147). Thus, in addition to promoting morphological changes in astrocytes that can modify the synaptic connectivity of metabolic circuits (10), leptin directly affects glutamate uptake by these glial cells, thus modulating excitatory neurotransmission. Leptin’s effects on glucose uptake by astrocytes (147) may also change the local transport of glucose and its metabolites to neurons, which would in turn modify glucose sensing mechanisms and neuronal control of energy homeostasis.

The physiological importance of the astrocyte-mediated effects of leptin was recently demonstrated by specific ablation of the leptin receptor in GFAP positive cells. In these mice, the synaptic organization of the melanocortin system is affected, as is the satiety response to leptin (132). Loss of the leptin receptor in GFAP positive cells decreased the number and length of astrocyte projections in the arcuate nucleus. This was associated with decreased astrocytic coverage of POMC neurons and an increase in the number of synaptic inputs to these neurons. Moreover, modifications in the electrical activity of both POMC and AgRP neurons were observed. These studies support the hypothesis that leptin’s effects on astrocytes are fundamental for the normal synaptic organization and synaptic transmission of metabolic circuits, as well as for mediating the physiological effects of this hormone. Furthermore, not only does loss of the leptin receptor in astrocytes decreases the physiological response to leptin, but it also increases the responses to ghrelin and to fasting (132).

Astrocytes express estrogen receptors (ERs) (148–150), androgen receptors, and progesterone receptors (151, 152), with gonadal steroid modulation of these glial cells possibly participating in the reported sexual dimorphism in the inflammatory response to HFD (153, 154). Males and females diverge in the manner that they store adipose tissue, with males being more prone to accumulate visceral fat, while females accumulate more subcutaneous fat. In addition, adipose tissue from males and females can also differ in its response to metabolic hormones (155, 156). Ingestion of a HFD increases systemic and hypothalamic palmitic acid levels. This rise in palmitic acid leads to an increase in the inflammatory cytokines IL1β, TNFα, and IL6 and a decrease in the anti-inflammatory cytokine IL10 in the hypothalamus of males, but not of females. Consequently, glucose tolerance and cardiovascular function are more affected in males than in females (154). Expression of ERα in astrocytes is a key factor in blunting the central inflammatory response to palmitic acid in females (154).

How other metabolic hormones and dietary components affect astrocyte functions remains to be demonstrated. For example, glucocorticoids modulate astrocytes in the suprachiasmatic nucleus (157) and whether this affects circadian rhythms of food intake remains to be determined. Ghrelin has direct effects on hypothalamic astrocytes (158), but how this affects metabolism is unknown. Fructose can induce astrogliosis (159), but intake of a high sucrose diet that stimulates adiposity does not induce classical astrogliosis (160); hence, further information is necessary regarding the effects of excess carbohydrates on astrocytes.

## Astrocytes in Development of Metabolic Circuits and Cell Turnover

Astrocytes play an essential role in neuronal survival and they most likely participate in the development of metabolic neuronal circuits. It is possible that astrocytes also mediate some of the detrimental effects of early environmental factors on long-term energy balance. For example, early neonatal overnutrition results in an increase in adult body weight, hyperleptinemia, and hyperinsulinemia, and these changes are associated with high hypothalamic GFAP levels (10). The rise in GFAP is at least partially due to an increase in the number of astrocytes in the arcuate nucleus, while astrocyte number was not affected in other hypothalamic areas (147). This suggests that the early nutritional environment modulates astrocyte development in the hypothalamus in an anatomically specific manner.

## Pathophysiological Role of Astrocytes in Metabolic Control

In addition to hypothalamic inflammation, HFD-induced obesity can also result in hypothalamic astrogliosis (8, 35, 42). Astrogliosis, or reactive astrocytosis, can be broadly defined as a change in the number of astrocytes and/or their morphology in response to an insult that is accompanied by a change in their release of growth factors, neurotrophic factors, cytokines, or other substances. Initially, the aim of this reaction is to defend and protect neurons and to produce modifications in the BBB in order to isolate the injured site and maintain the extracellular environment.

Activation of hypothalamic astrocytes can be seen as early as 24 h after HFD intake (8, 161). This rapid activation of astrocytes is most likely a neuroprotective response to the rise in the concentration of FAs reaching the CNS, as FAs cross the BBB by simple diffusion and in proportion to their plasma concentration (162–164). This acute astrocytic reaction to HFD intake appears to be involved in the mechanisms that are triggered in the attempt to maintain energy homeostasis (161).

Pathophysiological processes become apparent when astrocyte activation is prolonged and these glial cells begin to release neurotoxic substances and to form scar tissue (165). One situation that can lead to prolonged astrogliosis is obesity due to the long-term consumption of a HFD. LCFAs, such as palmitic acid, can directly induce the release of inflammatory cytokines, while unsaturated FAs, like oleic acid, do not appear to induce inflammation. This inflammatory response is mediated, at least in part, by FA activation of toll-like receptor 4 (35). Thus, one mechanism by which saturated FAs potentiate weight gain is through the direct stimulation of hypothalamic inflammation and the subsequent reduction in central leptin sensitivity (35).

Excess weight gain can result in an increase in the number of primary astrocytic processes, as well as in the length of these processes (10, 147). This increase in astrocytic processes can result in a more extensive contact between these cells and the local vasculature (9). These structural changes could thus modify the ability of astrocytes to transport substances from the circulation and into the brain. At the same time, increased glial coverage of hypothalamic neurons will result in less available space for these neurons to receive synaptic inputs. Indeed, synaptic reorganization in response to HFD-induced obesity results in the loss of synapses on POMC perikarya (9). Rapid synaptic changes may be involved in the metabolic adaptation to HFD intake (138) and be an attempt to regain energy homeostasis. In contrast, long-term changes could possibly contribute to produce a positive energy balance. Thus, although astrocyte activation in response to acute HFD may be involved in controlling the initial hyperphagia, long-term HFD causes increased energy intake and obesity (123).

## Astrocytosis in the Development of Insulin and Leptin Resistance

The inflammatory response of the hypothalamus to HFD-induced obesity can lead to decreased leptin and/or insulin sensitivity, which not only results in negative consequences in the regulation of food intake and energy balance, but also leads to secondary complications, such as type 2 diabetes (42, 166). Astrocytes contribute to this deregulation by not only producing inflammatory cytokines, but may also do so by modifying leptin and insulin availability. As stated above, HFD-induced obesity modifies the contacts between blood vessels and astrocytes (9). How these structural changes affect the transport of metabolites and hormones remains to be elucidated, but they could participate in modifications in leptin transport across the BBB (167–169). Circulating levels of leptin increase during obesity but transport into the brain does not increase proportionally, with the transport system becoming saturated (167). Expression of leptin receptors in astrocytes increases in the hypothalamus of obese rats (127, 130), but how this affects the output of metabolic circuits is not known at present.

## Oligodendrocytes

Oligodendrocytes are responsible for forming myelin sheaths around neuronal axons in the CNS. These glial cells have not been directly implicated in the control of food intake. However, recent studies have suggested a complex relationship between oligodendrocytes and other glial types, such as astrocytes (170) and microglia (171), in addition to their liaison with neurons. As Peferoen et al. (171) reviewed recently, there is evidence for an active role of these cells in inflammation. Moreover, an anti-apoptotic effect of ghrelin on oligodendrocytes during spinal cord injury has been reported and this may be mediated through inhibition of microglial activation (172, 173). Glucocorticoids can reduce the proliferation and viability of oligodendrocytes (174), while NPY and leptin have been shown to affect myelination by oligodendrocytes (175, 176). Even though direct involvement of oligodendrocytes in energy homeostasis has not yet been demonstrated, the above-mentioned studies suggest possible connections between these cells and metabolic control.

## Tanycytes

Tanycytes are specialized glial cells whose functions are increasingly linked to neuroendocrine control. They are found lining the third ventricle and can be classified into α1, α2, β1, and β2 from dorsal to ventral, with α1-tanycytes being found near the dorsomedial hypothalamic nuclei, and β2-tanycytes found close to the median eminence (ME) (177). These cells have long processes that project into the brain parenchyma or in the case of β2-tanycytes, processes that directly access the circulation through fenestrations of the BBB in the ME (177). These glial cells play a fundamental role in communicating the hypothalamus with systemic factors.

## Tanycytes in Sensing and Signaling

Tanycytes are able to detect changes in glucose levels. They express essential components of the glucose metabolism system used by pancreatic β-cells, such as GLUT-1, GLUT-2, glucokinase, and ATP sensitive K^+^ channels (178–180). Although this system in β-cells has a different functional significance (e.g., insulin release), evidence suggests that there is an ATP receptor-dependent mechanism through which tanycytes respond to changes in glucose levels (181). Selective destruction of tanycytes impairs the normal feeding response to hypoglycemia, and this is restored when these cells are regenerated (182), thus highlighting the importance of glucose metabolism in tanycytes for the control of energy balance.

It is thought that tanycytes detect the composition (i.e., glucose levels) of the CSF, rather than that of the extracellular fluid in the parenchyma (179, 183). In addition to glucose, these glial cells respond to non-metabolizable analogs of glucose and several transmitters, including histamine, acetylcholine, and ATP (184, 185). Like other glial cells, tanycytes produce Ca^2+^ signals that can propagate along the layer of these glial cells due to the gap junctions between them (185). They can release ATP and ADP and also express purinergic (P2) receptors that are activated in response to ATP. This increases the possibility of long-range activation via the release of ATP into the extracellular space, resulting in the activation of P2 receptors and triggering a new Ca^2+^ signal in neighboring tanycytes (181). Tanycytes also release lactate in response to glucose (186), with the lactate, ATP, and ADP released by these cells possibly affecting nearby hypothalamic neurons and modulating metabolism and appetite (180, 187, 188).

## Tanycytes as Gatekeepers

At the level of the ME, β-tanycytes form a blood-CSF barrier. The tight junctions between these tanycytes prevent the diffusion of blood-borne molecules into the CSF. As stated above, these cells have specialized processes that project into the fenestrated vessels of the ME (177). In a similar manner, α-tanycytes play an important role in the blood-arcuate nucleus interface, controlling the access of metabolic components to this brain area (189). In contrast, α-tanycytes do not have barrier properties (190, 191). Tight junctions at the apical bodies of α-tanycytes present a different composition and organization from those of β-tanycytes, preventing the α-tanycytes from forming a barrier at the level of the ventricular wall. Hence, endothelial cells perform the barrier properties at this blood-arcuate nucleus interface (191). Here, the long processes of the tanycytes are in close association with these vessels, suggesting that endothelial cell/tanycyte communication is of major importance in the organization of this interface (192).

Tanycytes express transporters for a variety of molecules, a fact that has lead researchers to suspect that these cells are involved in the process of transporting numerous substances into the brain (193). Balland et al. (194) have recently demonstrated that tanycytes act as a gateway for leptin into the brain, with this process requiring ERK-signaling. After its uptake by tanycytes, leptin then diffuses into the hypothalamic parenchyma to exert its effects (194) on neurons in the mediobasal hypothalamus and to reduce food intake.

Tanycytes also express deiodinase enzyme II (129, 195), which converts the prohormone T4 to the active hormone T3. They capture T4 from the circulation and liberate T3 to the surrounding hypothalamic nuclei, thus acting as gatekeepers for the entry of thyroid hormone into the hypothalamus (196). In the hypothalamus, T3 can regulate the response of NPY/AgRP neurons to fasting and facilitates rebound feeding (197).

In 2013, Langlet et al. (192) demonstrated that the tanycyte barrier is able to adapt to new metabolic conditions. Specific nutritional changes can modify the permeability of blood-hypothalamic barriers, allowing direct circulatory access to a subset of arcuate neurons. After 24 h of fasting, a drop in glucose levels, perceived by tanycytes themselves or possibly by astrocytes (198), triggers vascular endothelial growth factor (VEGF)-A expression in tanycytes that leads to the direct exposure, and consequently enhanced responsiveness, of a subset of neurons in the arcuate nucleus to peripheral metabolic signals (189). This, however, may not be a selective mechanism (198), and the possibility of this type of reorganization occurring in response to other types of energy imbalances (198) highlights the importance of this area of research.

These discoveries raise the question as to whether the tanycyte barrier is a gateway to the hypothalamus for other hormones and factors involved in energy balance. The ability of the nutritional environment to induce changes in this barrier is of great interest in order to further our understanding of long-term changes in metabolism. In addition, tanycyte-like cells bordering the circumventricular organs (CVOs) have been reported to share some features with ME tanycytes and display barrier properties. This indicates that tanycyte-like cells may be characteristic of CVOs (189) and may possibly be involved in other physiological functions.

Studies in seasonal mammals have increased our understanding of the tight relationship between tanycytes and energy balance. These animals experience photoperiodic adaptive changes in body weight and tanycytes express many of the proteins necessary for these adaptive changes, including the orphan receptor GPR50, the previously mentioned deiodinase, and a transporter for retinoic acid (181, 199). It should be noted that most of the above-cited studies were performed in murine models, and the functions of tanycytes in human physiology remain to be clearly demonstrated.

## Progenitor/Stem Cells

Even in adults there is a neurogenic niche in the hypothalamus that is sensitive to metabolic signals. However, the regenerative capacity of the hypothalamic neurogenic niche decreases with age and could contribute to age-associated weight gain. Different cell types can act as neural progenitors in this proliferative compartment. In the mediobasal hypothalamic parenchyma, populations of Sox2+ (200) and NG2+ cells (201) give rise to new neurons during adulthood. These new neurons express metabolic neuropeptides, such as POMC, NPY, AgRP, and orexin, and can respond to leptin and fasting (202). NG2+ cells are also known to be oligodendrocyte precursors (203–205) and could be performing this function in the hypothalamus (206).

Tanycytes also express proteins typically found in precursor cells (207) and are able to proliferate and differentiate into both neurons and glia (208). Some controversy exists regarding which group of tanycytes (α or β) is implicated in the adult hypothalamic niche. Some authors propose that these two precursor-cell subsets are functionally different, or that they may act sequentially with α-tanycytes dividing into β-tanycytes (202, 209–211). However, the fact that tanycytes are important in this process is now widely accepted.

In male mice, HFD reduces the constitutive turnover of neurons in the arcuate nucleus (212). This process is reversible by caloric restriction, which alone exerts the opposite effect (212). Hypothalamic neuronal precursor cells (NPCs) can be damaged by HFD-induced inflammation, and upregulation of inflammatory mediators IKKB/NF-κB inhibits the proliferation and survival of adult NPCs in the hypothalamus. Furthermore, neurogenesis can be inhibited and obesity induced by selective over-expression of IKKB in hypothalamic NPCs in adult male mice on a normal diet (200). Importantly, it seems that some of the effects of HFD are sexually dimorphic in the ME, promoting neurogenesis in young adult female mice but not in males (213). Adult-born neurons in the ME are in part responsible for weight gain in female mice (213). Several recent studies also relate HFD or energy balance alterations to changes in neurogenesis and remodeling of feeding circuits in the hypothalamus (207, 211, 214), although the specific involvement of tanycytes or other glial cells was not investigated.

## Ependymal Cells

Ependymal cells or ependymocytes are classified as macroglia and are derived from the neuroectoderm. The majority of these cells are born during the embryonic and early postnatal periods in most species (215, 216). Ependymal cells line the ventricles throughout the brain, including the dorsal part of the third ventricle next to the hypothalamus. There is a transitional zone at the middle of the third ventricle where both ependymal cells and tanycytes can be found, with only tanycytes existing in the ventral zone of the third ventricle (217, 218). The morphology of ependymocytes is very characteristic as they have numerous cilia, although ependymal cells with a long basal body and only two cilia have been described in the lateral ventricular zone (217). These cells secrete CSF, with their numerous cilia participating in the transport of this fluid.

During critical periods of development, third ventricle ependymal cells express receptors for important metabolic hormones suggesting that they may participate in the transport of metabolites and signals necessary for correct development. For example, ependymal cells lining the third ventricle express receptors for the satiety-inducing peptide cholecystokinin (CCK-1) early in life, with this expression occurring at critical developmental stages (219). Expression of the CCK-1 receptor peaks at postnatal day 6 but is undetectable at postnatal day 12. On the contrary, expression of the CCK-2 receptor in ependymal cells does not appear to depend on the developmental stage. Expression of the leptin receptor in ependymal cells of the third ventricle is also transient. These cells express functional leptin receptors early in life, with the level of expression increasing at specific times during the development of metabolic circuits. These receptors then disappear later in development, approximately at the same time that the ependymal layer becomes thinner (220).

Ependymal cells are present in neurogenic regions and have a key function regarding the formation/genesis and migration of new neurons and glial cells (217), but it was only recently demonstrated that neurogenesis in the hypothalamus plays an important role regulating energy balance(211). As mentioned above, obesity and HFD intake can decrease neurogenesis in the adult hypothalamus and this leads to deregulation of metabolic control. In addition to tanycytes, ependymal cells surrounding the third ventricle form part of the hypothalamic neurogenic niche (207). However, the neurogenic response of ependymal cells and tanycytes may differ, as tanycytes are reported to proliferate in response to IGF-1, while ependymal cells do not (218).

## Endothelial Cells

Endothelial cells are derived from the mesoderm and form the lining of the brain vasculature and thus comprise an integral component of the BBB. Endothelial cells of hypothalamic blood vessels have been demonstrated to participate in metabolic control. Indeed, changes in hypothalamic vasculature and endothelial cell morphology have been observed in response to a high fat or a high sucrose diet (221), not only in rodent models but also in human patients with type 2 diabetes. Likewise, low glucose levels as a consequence of fasting have been shown to regulate BBB permeability and tanycyte reorganization (189), with tanycytes and hypothalamic microvessels exhibiting an adaptive response to changes in the general metabolic status and thus, participating in glucosensing.

## Concluding Remarks

Non-neuronal cells are involved in all aspects of brain function. However, much is yet to be learned as to how these cells are modulated by both central and systemic factors and how their output or response to these signals affects neighboring neurons. The fact that HFD-induced obesity was shown to produce some of its adverse metabolic effects through activation of hypothalamic glial cells has led to an increased interest in this process. However, in addition to this pathological function, it is now apparent that non-neural cells are also fundamental in the physiological control of metabolism. As we know more about how these cells participate in the control of food intake, the discovery of new therapeutical approaches to metabolic diseases, such as diabetes, obesity, or leptin resistance, looks increasingly plausible. For example, studies demonstrating that tanycytes are responsible for leptin transport into the brain suggest that they may be a potential therapeutic target for the treatment of leptin resistance in obesity through increasing their transport of this hormone into the hypothalamus. Discovery of ways to protect against inflammatory processes that can damage the hypothalamus, as well as to stimulate progenitor cells in order to renovate metabolic circuits is of clear interest for the future. Moreover, it is now clear that all investigators interested in the cellular mechanisms controlling appetite and metabolism should understand how glial cells participate in this process.

## Conflict of Interest Statement

The authors declare that the research was conducted in the absence of any commercial or financial relationships that could be construed as a potential conflict of interest.
